# Primary Pyomyositis of the Adductor and Pectineus Muscle in Patients With Uncontrolled Diabetes: Report of Two Cases

**DOI:** 10.1155/cro/6657339

**Published:** 2026-04-21

**Authors:** Shuichi Miyamoto, Yuichi Yoshii, Kazumasa Watanabe, Toshinori Tsukanishi, Kentaro Mataki, Toru Uchida, Tomomi Suzu, Reo Asai, Tomoo Ishii, Daisuke Himeno

**Affiliations:** ^1^ Department of Orthopedic Surgery, Tokyo Medical University Ibaraki Medical Center, Ami-machi, Ibaraki, Japan, tokyo-med.ac.jp; ^2^ Department of Orthopedic Surgery, Chiba Emergency Medical Center, Chiba City, Chiba, Japan

## Abstract

Muscle amyotrophy and infarction are known to be rare complications of patients with diabetes mellitus and poor glycemic control. However, primary pyomyositis, an especially rare infection of the primary muscles, is also a problem that cannot be ignored. Primary pyomyositis typically begins as a subacute condition and is probably due to transient bacteremia. Here, we report on two patients with Type 2 diabetes mellitus and poor glycemic control who developed primary pyomyositis, which manifested as abscesses of the adductor and pectineus muscles. Both patients underwent surgical drainage, and one patient additionally underwent internal fixation while receiving a continuous local antibiotic perfusion. Both patients were well and without signs of recurrence at their final follow‐up visit. Primary pyomyositis, in addition to muscle amyotrophy and infarction, should be included in the differential diagnosis of infections occurring in patients with painful lesions in the lower extremities and Type 2 diabetes mellitus with poorly controlled glycemia.

## 1. Introduction

Primary pyomyositis is a bacterial infection of the musculoskeletal system that is characterized by the formation of abscesses. It is thought to be associated with transient bacteremia [1–4]. A previous large matched cohort study that used the Canadian Primary Care Sentinel Surveillance Network found that the incidence of infections in the musculoskeletal regions, including those in the skin and soft tissue, was 37.9%, which is similar to the incidence of other types of infections [5]. This condition is a well‐recognized clinical entity in tropical regions, particularly in Africa, Southeast Asia, and South America [6–8]. However, an increasing number of cases have also been reported from temperate regions, including Japan [3, 9–12]. Among the various underlying conditions associated with primary pyomyositis, diabetes mellitus is one of the most important contributing risk factors [3, 11, 13]. In Japan, the National Health and Nutrition Survey performed a study of 10 million people from 2009 to 2019 and found that 19.7% of men and 10.8% of women in the study had conditions that were strongly suspected to be diabetes mellitus and that those proportions had increased over the survey period [14].

Patients with diabetes mellitus generally have an increased susceptibility to infections [15–17]. However, musculoskeletal complications associated with diabetes are relatively uncommon. Among these conditions, muscle amyotrophy and diabetic muscle infarction have been documented [18, 19], and primary pyomyositis, an especially rare infection of the primary muscles, is also a problem that cannot be ignored.

Here, we report the cases of two patients with Type 2 diabetes mellitus and poor glycemic control who had primary pyomyositis with abscesses in the adductor and pectineus muscles. We found that it was possible to salvage limbs successfully without amputation by performing open surgical drainage and internal fixation and administering antibiotic therapy via a continuous perfusion of local antibiotics (CLAP).

## 2. Case Presentation

Informed consent to allow publication was obtained from each of the two patients described in this report.

### 2.1. Case 1

**Figure 1:**
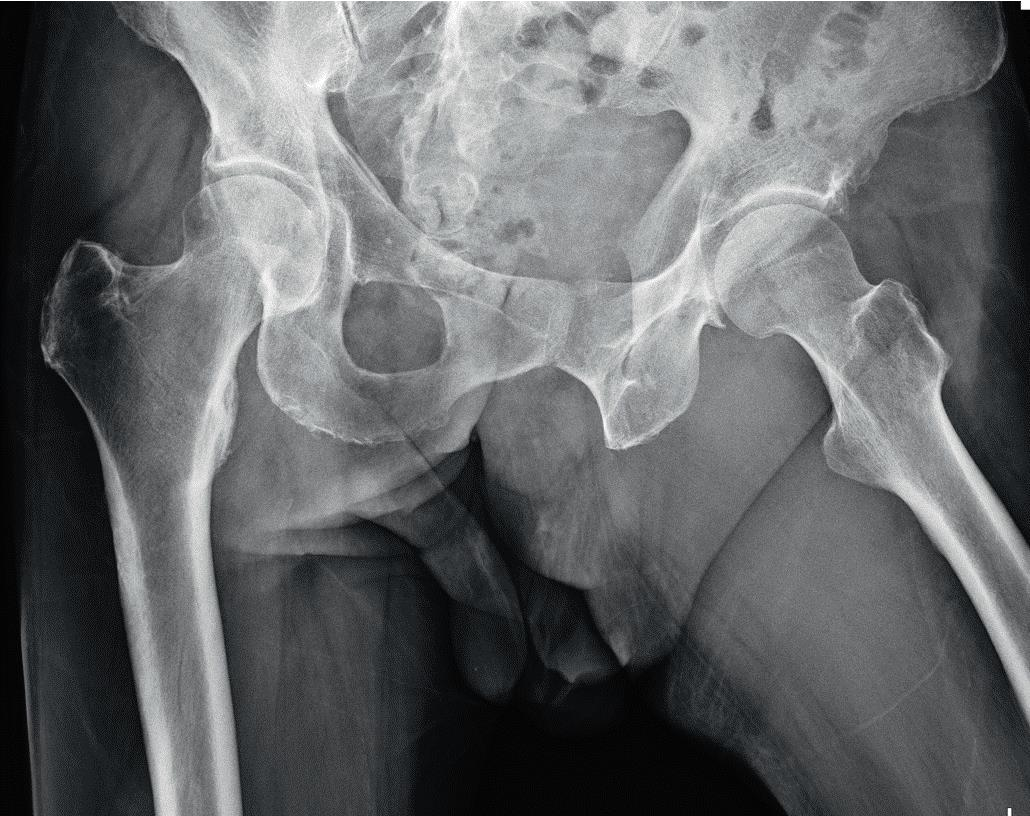
Case 1. Preoperative anteroposterior radiographs of bilateral hip joints.

**Figure 2:**
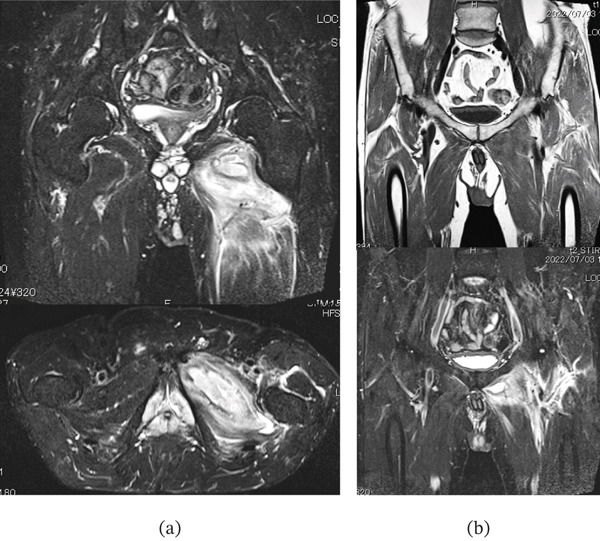
Case 1. (a) Preoperative coronal and axial hip MRI with short T1 inversion recovery (STIR) images and (b) coronal MRI with T1‐weighted imaging and STIR images of the lower lumbar spine and the hip.

A 48‐year‐old man was admitted to our hospital because of a 1‐week history of pain in his left proximal thigh, fever, and generalized fatigue. His past medical history was remarkable for 10 years of untreated hyperglycemia. Physical examination revealed swelling and tenderness around the adductor muscles. There were no wounds, episodes of traumatic injury, or signs of psoas involvement. The relevant abnormal laboratory data at the time of admission were highly elevated levels of C‐reactive protein, white blood cells, and hemoglobin A1c (Table 1). Radiography revealed a left hip joint that was extended, externally abducted, and rotated (Figure 1). Magnetic resonance imaging (MRI) revealed a fluid collection in the left adductor brevis and pectineus muscles (Figure 2a). The L4 and L5 segments of the iliopsoas muscle were normal (Figure 2b). On admission, a drainage tube was placed after open debridement and irrigation were performed, with the patient under general anesthesia. Staphylococcus aureus was subsequently detected in both the bacterial culture of the abscess fluid and the blood culture. After surgery, the patient was treated with intravenous cefazolin (CEZ) sodium (1 g every 8 h) for 2 weeks, followed by oral ampicillin (AMP) (125 mg every 6 h) for another 8 weeks, and his blood glucose levels were carefully controlled. The drainage tube was removed on the 17th day after open drainage. The patient′s postoperative recovery was uneventful, and he was discharged from the hospital 3 weeks after the surgery.

**Table 1:** Laboratory and urinalysis findings on hospital admission.

	Patient results	
	Case 1	Case 2
Blood cell counts		
White blood cells (/*μ*L)	12.7 × 10^3^	13.7 × 10^3^
Neutrophils (%)	84.9	91.5
Lymphocytes (%)	6.8	3.1
Monocytes (%)	8.0	4.3
Eosinophils (%)	0.1	0.7
Basophils (%)	0.2	0.4
Red blood cells (/*μ*L)	4.16 × 10^6^	4.32 × 10^6^
Hemoglobin (g/dL)	11.9	13.4
Hematocrit (%)	35.7	38.3
Platelet (/*μ*L)	304 × 10^3^	484 × 10^3^
ESR (mm/h)	104	85
Laboratory tests		
Total protein (g/dL)	7.0	5.8
Albumin (g/dL)	2.3	2.6
Aspartate aminotransferase (IU/L)	25	14
Alanine transferase (IU/L)	28	15
Blood urea nitrogen (mg/dL)	9.1	23.4
Creatinine (mg/dL)	0.52	0.52
Uric acid (mg/dL)	3.6	5.6
Na (mg/dL)	130	126
K (mg/dL)	4.1	4.1
Cl (mg/dL)	91	91
Glucose (mg/dL)	371	398
HbA1c‐NGSP (%)	12.9	9.6
Immunochemistry		
CRP (mg/dL)	28.47	30.76
PCT (ng/mL)	0.16	2.09
Urinalysis		
Specific gravity	1.048	1.010
pH	6.0	5.5
Glucose (mg/dL)	2000	2000
Protein (mg/dL)	100	30
Ketones	2+	2+

Abbreviations: CRP, C‐reactive protein; ESR, erythrocyte sedimentation rate; HbA1c, hemoglobin A1c; NGSP, National Glycohemoglobin Standardization Program; PCT, procalcitonin.

**Figure 3:**
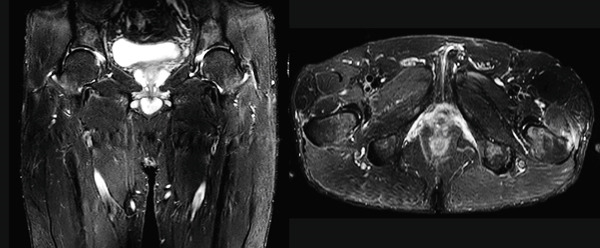
Case 1. One year after surgery, coronal and axial MRI with STIR images of bilateral hips and femurs.

At the final follow‐up 1 year after surgery, the patient′s signs/symptoms such as pain and swelling of the thigh were absent, and the laboratory data were normal. MRI confirmed complete resolution of the abscess (Figure 3). Function was assessed using the Japanese Orthopedic Association hip score (JOAHS) [20] and the modified Harris Hip Score (HHS) [21]. JOAHS assessment was as follows: pain = 35 points, range of motion = 20 points, walking = 20 points, and activities of daily living = 18 points, for a total of 93 points. The modified HHS assessment was as follows: pain = 40 points, limping = 11 points, support = 11 points, distance walked = 11 points, stairs = 2 points, shoes/socks = 4 points, sitting = 5 points, and public transportation = 1 point, for a total of 85 points.

### 2.2. Case 2

**Figure 4:**
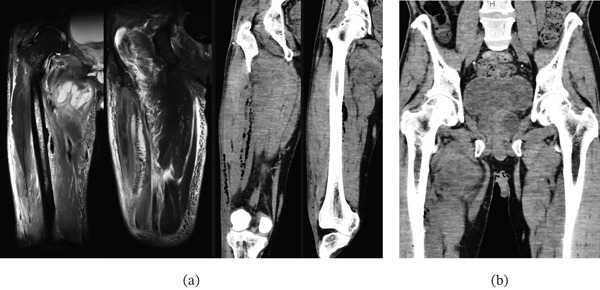
Case 2. (a) Preoperative coronal MRI with STIR image of the right hip and femur, a coronal CT image of the right hip and femur, and (b) a coronal CT image of the lower lumbar spine and hip.

A 58‐year‐old man visited our hospital because of a 2‐week history of increasing pain in the medial proximal area of his right femur, along with fever and difficulty walking. His medical history was notable for a 20‐year history of treated Type 2 diabetes mellitus, with a hemoglobin A1c level consistently greater than 8.0% for the past year. Physical examination revealed diffuse swelling in the right thigh with tenderness around the adductor muscle, but no signs of inflammation in the iliopsoas muscle. Laboratory examination at the time of admission showed highly elevated levels of inflammatory markers and hemoglobin A1c (Table 1). MRI and computed tomography (CT) revealed evidence of gas within a fluid collection extending from the right adductor brevis and pectineus muscles to the vastus lateralis and biceps femoris muscles (Figure 4a) and no evidence of swelling or fluid collection within the iliopsoas muscle (Figure 4b). The patient underwent open surgical drainage twice under general anesthesia: once upon admission and 1 week after admission. Klebsiella pneumoniae was detected in both the bacterial culture of the drainage fluid and the blood culture. After admission, intravenous CEZ (1 g every 8 h) was administered for 5 weeks, followed by an additional 3 weeks of oral levofloxacin (500 mg every day), in conjunction with correction of his hyperglycemia. The drainage tube was removed 3 weeks after the second surgical drainage. The patient was discharged from the hospital after 12 weeks with normal levels of inflammatory markers.

**Figure 5:**
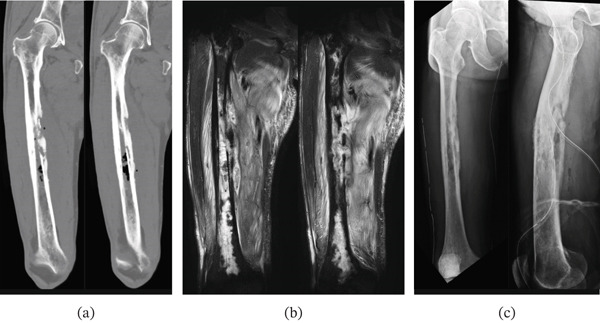
Case 2. (a) Preoperative coronal CT image of the right hip and femur at readmission, (b) coronal MRI with STIR image of the right hip and femur at readmission, and (c) postoperative anteroposterior and lateral radiographs of the right hip and femur.

**Figure 6:**
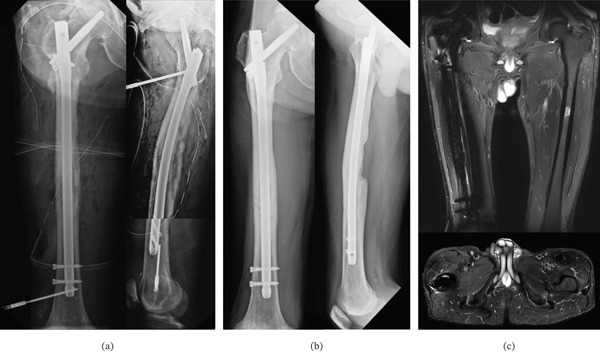
Case 2. (a) Anteroposterior and lateral radiographs of the right hip and femur after internal fixation with an intramedullary femoral nail combined with continuous local antibiotic perfusion (CLAP), (b) anteroposterior and lateral radiographs of the right hip and femur taken 6 months after internal fixation with CLAP, and (c) coronal and axial MRI with STIR images of bilateral hips and femurs taken 6 months after internal fixation plus CLAP.

At 8 weeks after his discharge, the patient was readmitted with increasing pain and swelling in his right thigh and inability to walk. CT revealed cortical bone destruction, periosteal reaction, osteolysis of the medial cortex of the femoral diaphysis, and intraosseous gas within the medullary canal of the distal femur (Figure 5a). MRI revealed high signal intensity in the adductor brevis, longus, and magnus muscles, as well as a fluid collection around the medial cortical defect of the femoral diaphysis on the short T1 inversion recovery image (Figure 5b). Open surgical debridement and irrigation, with insertion of a drain, were performed upon readmission of the patient (Figure 5c). Klebsiella pneumoniae was detected in the intraoperative fluid, including a specimen from the abscess. Based on these findings, a diagnosis of impending fracture of the femoral diaphysis with osteomyelitis caused by the recurrence of abscesses in the adductor and pectineus muscles was made. Internal fixation with an intramedullary nail (TRIGEN INTERTAN 13 × 360mm; Smith & Nephew) combined with CLAP was performed 2 weeks after readmission (Figure 6a). Intramedullary antibiotic perfusion was continued for 2 weeks via two bone marrow needles, and antibiotic perfusion into the soft tissue was carried out for 4 weeks via two dual‐lumen tubes. Gentamicin (1.2 mg/mL) was infused through these needles and tubes at a rate of 2 mL/h throughout that period. Intravenous antibiotic treatment was started with CEZ (1 g every 8 h) and continued for 6 weeks postoperatively. The patient then received oral antibiotic therapy with sulfamethoxazole/trimethoprim (800mg/160mg every 12h) for an additional 3 weeks. Three weeks postoperatively, laboratory markers of inflammation revealed normal levels, and the patient was discharged 8 weeks after readmission. One year postoperatively from the first course of CLAP, a second course of CLAP was administered to the patient because of the recurrence of an abscess on the posterolateral aspect of the vastus lateralis muscle and osteomyelitis of the distal femur.

Six months after internal fixation and CLAP, follow‐up x‐rays showed evidence of bone remodeling in the femoral diaphysis at the fracture site (Figure 6b). MRI showed that the abscesses in the adductor brevis and pectineus muscles had essentially disappeared, with normal signal intensities (Figure 6c), and the levels of inflammatory markers were normal. JOAHS assessment was as follows: pain = 35 points, range of motion = 18 points, walking = 20 points, and activities of daily living = 18 points, for a total of 91 points. The modified HHS assessment was as follows: pain = 40 points, limping = 8 points, support = 11 points, distance walked = 11 points, stairs = 2 points, shoes/socks = 4 points, sitting = 5 points, and public transportation = 1 point, for a total of 82 points.

## 3. Discussion

We described two patients with Type 2 diabetes mellitus and poor glycemic control who developed primary pyomyositis with abscesses in their adductor and pectineus muscles. Although abscesses in the adductor and pectineus muscles are rare, they should be considered in the differential diagnosis of the primary focus of infection in patients with Type 2 diabetes mellitus and poor glycemic control. Furthermore, prompt surgical intervention combined with adequate antibiotic therapy should be initiated as soon as possible after confirmation of an abscess, with the goal of improving survival outcomes and salvaging the lower limbs.

The original descriptions of primary pyomyositis have long been referred to as tropical pyomyositis or myositis tropicans, as the condition was historically predominantly reported from tropical regions, particularly Africa, Southeast Asia, and South America [6–8]. Pyomyositis is divided into three clinical stages [3, 8]. The first stage, known as the invasive stage, during which the affected muscle becomes inflamed and painful, is characterized by a subacute onset with variable fevers, firm and tender swellings, and minimal systemic symptoms. The second suppurative stage, which typically occurs 10–21 days after the first stage, is characterized by the formation of a muscle abscess, during which patients usually experience severe pain, progressive swelling, and fever. The majority of patients are diagnosed during this stage [8]. In the third stage, the muscle tissue contains a large, often multifocal abscess. At this point, patients frequently exhibit systemic toxicity, which may progress to sepsis. Staphylococcus aureus is the predominant causative organism regardless of geographic region [3].

Pyomyositis in tropical regions occurs across all age groups and has been reported to be particularly common in children [22] and in individuals aged 20–45 years [23]. Traditionally, it had been characterized as occurring in previously active and healthy young men [3]. There have been increasing numbers of reports of pyomyositis from temperate regions, including Japan [3, 9–12], where they have primarily been described in hosts with impaired immunity—such as those with diabetes mellitus, human immunodeficiency virus (HIV) infection, hematologic malignancies, or chronic kidney disease—or in individuals with compromised general health due to other underlying conditions [3, 6, 13]. A recent systematic review based on studies largely conducted in tropical regions has demonstrated a significant association between pyomyositis and both HIV infections and acquired immunodeficiency syndrome (AIDS). Therefore, some cases of tropical pyomyositis may be associated with backgrounds such as HIV infections, AIDS, or parasitic diseases [1].

In temperate regions, there have also been cases of pyomyositis reported in children and otherwise healthy individuals without underlying comorbidities [24–26]. Travel to or migration from tropical areas may contribute to the onset of disease [22]. Therefore, apart from the fact that the most predominant causative pathogen is Staphylococcus aureus [6], differentiation between the manifestations of pyomyositis occurring in tropical versus temperate regions has become increasingly difficult [3].

In Japan, primary pyomyositis has now been reported across an age spectrum ranging from infancy to older adults, which is similar to the age spectrum of the disease in tropical regions [27–29]. Many older adults have developed the disease in their 70s–80s, and affected patients frequently have comorbid conditions such as diabetes mellitus, solid tumors being treated with chemotherapy, hematologic malignancies, or metabolic bone disease [29–32]. Additionally, as noted previously, similar findings have also been reported in otherwise healthy individuals [33, 34]. Consistent with the global experience, Staphylococcus aureus is the most frequently reported pathogen in Japanese cases of primary pyomyositis [30, 35, 36].

Previous studies have identified several predisposing conditions associated with an increased risk of primary pyomyositis; among these, diabetes mellitus is recognized as one of the most significant clinical determinants [3, 11, 13]. Type 2 diabetes mellitus associated with infection is strongly related to hyperglycemia [37].

Hyperglycemia has played a major role in the pathophysiology of innate immune dysfunction in vivo, particularly affecting manifestations of innate cellular immunity such as chemotaxis, phagocytosis, and the bactericidal activity of neutrophils and macrophages [38–41]. Furthermore, it is well known that hyperglycemia and high levels of urinary glucose encourage bacterial growth [42, 43]. It is also known that both microangiopathy and neuropathy, which are induced by diabetes, contribute to the development of infections [44–46].

Amyotrophy and infarction are regarded as complications of musculoskeletal disorders in patients with diabetes mellitus [18, 19]. On the other hand, the association between pyomyositis and diabetes mellitus also cannot be ignored [11, 13].

The underlying pathogenesis of primary pyomyositis has not yet been clearly elucidated. Primary pyomyositis is a suppurative infection of striated muscle, which is presumed to result from hematogenous seeding during episodes of transient bacteremia [1–4]. A prestigious epidemiological review of 676 patients with primary pyomyositis found that pyomyositis of the adductor muscles was rare, with an incidence reported at 2.8% in terms of the anatomic distribution of the condition [2]. A previous report on abscesses of the adductor muscles described their association with iliopsoas abscesses and osteomyelitis of the symphysis pubis [47–50]. As there was no convincing evidence of inflammation in either the iliopsoas muscles or symphysis pubis in our cases, we believe that pyomyositis with abscesses in the adductor and pectineus muscles occurred as a primary infection following transient bacteremia. Previous reports have suggested that the initial signs and symptoms of muscle infarctions in diabetes mellitus occur more frequently in the thigh region and there is an association between muscle infarctions and the adductor muscles [51–54]. These findings lead us to speculate that muscle infarctions in diabetic patients may contribute to the pathogenesis of pyomyositis.

According to previous reports, osteomyelitis and septic arthritis have been documented relatively frequently as complications of pyomyositis [55, 56]. In Case 2, the extensive spread of the infection resulted in osteomyelitis, which was difficult to manage. In addition, it should be recognized that the occurrence of a pulmonary embolism secondary to deep vein thrombosis associated with prolonged immobilization or severe systemic inflammation, as well as septic shock, represents potentially fatal complications of pyomyositis [57, 58].

Regarding the standard treatment protocol for pyomyositis, the prompt drainage of abscesses—performed under ultrasound or CT guidance or through surgical intervention—along with rapid identification of the bacterial source of infection and intravenous administration of a broad‐spectrum or adequate antibiotics, has important implications for improving survival rates as well as for salvaging the lower limbs and preserving their function [2, 3]. An important consideration is that the mortality rates, ranging from 1% to 20%, are by no means low; therefore, once the disease is suspected, therapeutic intervention should be initiated as early as possible [1, 6, 8, 11].

CLAP was recently introduced as a novel therapeutic strategy for osteomyelitis and soft tissue infections, and its effectiveness has been demonstrated clinically [59, 60]. CLAP combined with internal fixation was considered effective, especially for Case 2, which was complicated by an impending fracture due to osteomyelitis, as it improved mechanical stability and increased the local concentration of antibiotics. This technique has been a useful and effective surgical treatment for pyomyositis with osteomyelitis in Case 2.

## 4. Conclusion

We presented two cases of primary pyomyositis manifesting as abscesses of the adductor and pectineus muscles in patients with Type 2 diabetes mellitus and poor glycemic control. Treatment requires early surgical intervention as soon as the diagnosis is confirmed, along with effective antibiotic therapy targeting the causative organisms. CLAP combined with internal fixation is a useful surgical treatment option in cases involving osteomyelitis with an impending fracture.

## Author Contributions

S.M. collected and curated the clinical images and data and contributed to the drafting of the manuscript. Y.Y. contributed to manuscript editing, clinical interpretation, and outpatient follow‐up. K.W. was responsible for the surgical management of the case and for the clinical follow‐up of the patient during hospitalization. T.T. provided methodological support. K.M. was responsible for the collection of clinical data and images. T.U. participated in the surgical procedure, organized the clinical images, and contributed to data curation. T.S. participated in the surgical procedure and conducted clinical follow‐up during hospitalization. R.A. participated in the surgical procedure. T.I. provided technical supervision. D.H. contributed to the preoperative surgical planning and participated in the surgical procedure.

## Funding

No funding was received for this manuscript.

## Disclosure

All authors have read and approved the final manuscript.

## Consent

Written informed consent was obtained from both patients for the publication of this case report and any accompanying clinical data and images.

## Conflicts of Interest

The authors declare no conflicts of interest.

## Data Availability

All relevant clinical data supporting the findings of this case report are included within the article. Additional details are available from the corresponding author upon reasonable request, in accordance with patient confidentiality.
